# Donafenib treatment for hepatocellular carcinoma: A case report

**DOI:** 10.1097/MD.0000000000026373

**Published:** 2021-06-25

**Authors:** Qiaoqi Li, Hong Zhu

**Affiliations:** Department of Medical Oncology, Cancer Center, West China Hospital, Sichuan University, Chengdu, Sichuan, P.R. China.

**Keywords:** case report, deuterium-containing drug, donafenib, hepatocellular carcinoma

## Abstract

**Rationale:**

Hepatocellular carcinoma (HCC) is the most common liver cancer. The efficacy of the present treatment is disappointing, and the prognosis is poor. Donafenib, a novel multikinase inhibitor, is a new deuterated derivative of sorafenib. It can improve overall survival in patients with advanced HCC, with a favorable safety and tolerability profile over sorafenib.

**Patient concerns:**

Here, we report the case of a 51-year-old male patient who presented with experienced epigastric discomfort for the prior several days. He had a history of untreated chronic hepatitis B virus infection for >29 years and no other underlying diseases. Based on further investigations, he was diagnosed with advanced HCC and refused surgery.

**Diagnosis:**

Based on the patient's performance status, tumor status assessed by computed tomography, liver function, and percutaneous liver biopsy, he was diagnosed with advanced HCC Barcelona Clinic Liver Cancer Stage C.

**Interventions:**

The patient was administered a 200-mg oral dose of donafenib twice-daily.

**Outcomes:**

The patient was followed-up from the time of diagnosis. He received donafenib for 31 months, and the progression-free survival time was 31 months (from May 2017 to December 2019); the overall survival time was not reached. The patient reported little abdominal distension with no other obvious discomfort while taking the medication.

**Lesson:**

Donafenib showed good efficacy for the treatment of advanced HCC, with mild side effects. Deuterium-containing drugs seem to be a promising avenue for medical innovation.

## 1 Introduction

Primary liver cancer is a common malignancy of the digestive tract worldwide. According to the latest data published in the GLOBOCAN database in 2018, there were 841,080 new cases and 781,631 deaths of liver cancer, ranking it as the sixth most commonly diagnosed malignancy, and the fourth cause of cancer-related mortality globally.^[1]^ Hepatocellular carcinoma (HCC) is the most common pathological type of primary liver cancer, accounting for 85% to 90%.^[2,3]^ In China, the incidence of liver cancer is particularly high and hepatitis B-related HCC accounted for 70%. More than 60% of newly diagnosed patients are at an advanced stage when they are no longer eligible for surgery.^[2]^ HCC is resistant to traditional chemotherapy.^[4,5]^ However, the development of targeted drugs, such as small molecule tyrosine kinase inhibitors, has extended the overall survival (OS) of a subset of patients with advanced HCC. Sorafenib has been the standard first-line therapy for patients with advanced HCC for more than a decade, since the Sorafenib Hepatocellular Carcinoma Assessment Randomized Protocol and Oriental studies. However, its use is constrained by the side effects.^[6,7]^

Donafenib, a novel multikinase inhibitor, is a new patent drug that substitutes the hydrogen (H) of the methyl (-CH3) on the sorafenib amide bond with deuterium (D).^[8]^ It has been reported to improve OS in advanced HCC with a favorable safety and tolerability profile over sorafenib.^[9,10]^ Here, we report the case of a patient with advanced HCC treated with donafenib who attained an impressively long progression-free survival and OS. This study was approved by the Ethics Committee of West China Hospital of Sichuan University, and written informed consent was obtained from the patient.

## 2 Case presentation

**Figure 1:**
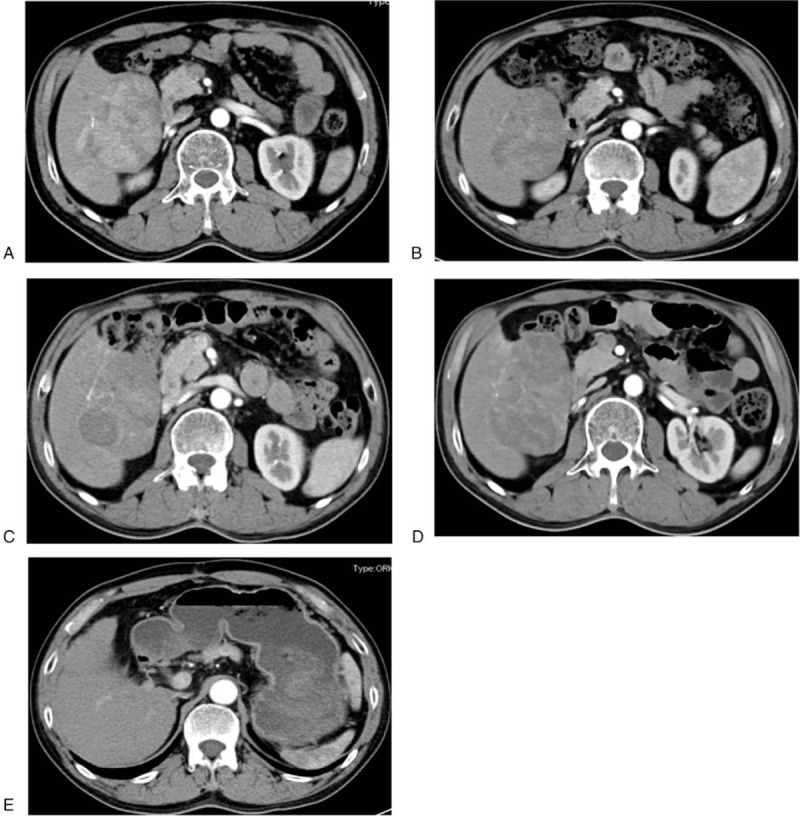
(A) The liver before donafenib treatment (May 23, 2017), the longest tumor diameter is 7.94 cm. (B) The liver after 12 months of donafenib treatment (June 20, 2018), the longest tumor diameter is 7.47 cm. (C) The liver after 24 months of donafenib treatment (May 21, 2019), the longest tumor diameter is 7.99 cm. (D) The liver after about 31 months of donafenib treatment (December 17, 2019), the longest tumor diameter is 8. 19 cm. (E) The liver after radical surgery (June 8, 2020), the patient remained cancer-free.

In May 2017, a 51-year-old man presented with epigastric discomfort for the previous several days. He had a history of untreated chronic hepatitis B virus infection for >29 years. He reported no other underlying diseases. At presentation, his body mass index was 22.0 kg/m^2^ (weight, 60 kg; height, 165.0 cm). He underwent further diagnostic investigations. A computed tomography (CT) scan showed 1 target lesion located in the right hepatic lobe, measuring up to 7.94 × 5.83 cm (Fig. 1A), with the characteristic radiological appearance of HCC (hyperenhancement during the arterial phase and delayed washout in the venous phase); no extrahepatic metastases and no portal vein invasion were detected. Laboratory tests results showed unusually elevated basal serum alpha-fetoprotein levels (>1210 μg/*L*). The patient had no ascites or hepatic encephalopathy. Laboratory tests showed that his total bilirubin <34 umol/L, albumin >35 g/ L. His coagulation function was normal. The Child-Pugh score was 5 (level A). Based on the performance status (Eastern Cooperative Oncology Group scale, 1), tumor status, and liver function, he was classified as having Barcelona Clinic Liver Cancer Stage C HCC. In addition, the patient underwent percutaneous liver biopsy for further diagnosis. Consequently, the patient was diagnosed with HCC clinically and pathologically.

According to the HCC guidelines, the patient was eligible for radical surgery.^[11]^ However, he adamantly refused the surgery and chose targeted therapy instead. Approximately 10 days after diagnosis, the patient began to take entecavir as antiviral treatment and donafenib (200 mg orally twice daily) as anti-tumor therapy (starting on May 26^th^, 2017). During the treatment, laboratory tests assessing liver function, coagulation, peripheral blood cells, and urine were performed every 4 weeks. Adverse effects were assessed using the Common Terminology Criteria for Adverse Events version 5.0. A CT scan was performed every 8 weeks.

After 8 weeks of treatment, the longest diameter of the target lesion decreased to 7.57 cm, and the alpha-fetoprotein level was stable. At 24 weeks of treatment, the longest diameter of the target lesion reached the lowest value (7.23 cm). The Response Evaluation Criteria in Solid Tumors Version 1.1 were used to determine the treatment efficacy and oncological response, which was stable disease (Fig. 1A–C). After 31 months of continuous therapy, the disease progressed (progression-free survival time was 31 months, from May 2017 to December 2019) (Fig. 1D). During the treatment period, the patient had persistent hand-foot syndrome (highest level 2), intermittent diarrhea (level 2), thrombocytopenia (level 2), hypertension (level 2–3), hypophosphatemia (level 2), and other adverse events. Except for hypophosphatemia, which was only kept under observation, all other adverse events gradually relieved after symptomatic treatment and dose adjustment of donafenib was not required. After disease progression in December 2019, the patient underwent radical surgery. A recent postoperative CT scan from June 2020 (Fig. 1E) showed that he remained cancer-free.

## 3 Discussion

Here, we reported a patient with advanced HCC had an impressive survival achieved without surgery. He administered the relatively low dose of donafenib for a long period of time without experiencing severe adverse effects. Donafenib pointed a promising choice for patients with advanced HCC because of the favorable safety and tolerability.

In 2017, the US FDA approved the first deuterium-containing drug, deutetrabenazine, a tetrabenazine derivative.^[12]^ Deuterium–carbon bonds are stronger than hydrogen–carbon bonds, leading the molecule more stable against metabolism by enzymes such as cytochrome P450. Cytochrome P450 2D6 is the most important enzyme for drug metabolism. As a result, the deuterated drug is metabolized more slowly than the regular one. When compared to tetrabenazine, the frequency of administration and dosage of deuterium-substituted derivatives are lower, leading to lighter adverse effects. Another advantage is that they are easily synthesized. According to the published literature, there are >10 ways to synthesize deuterium-containing drugs, which means that they can be produced on a kilogram scale.^[12,13]^ Many pharmaceutical producers have started switching toward the deuterated form of drugs. A new era of deuterated drugs has begun.^[14–16]^

Donafenib is strongly related to sorafenib. It is a deuterium derivative of sorafenib produced by substituting the hydrogen (H) of the methyl (-CH3) on the sorafenib amide bond with deuterium (D).^[8]^ The improvement in safety is probably due to the introduction of deuterium (D), which reduces the rate of drug metabolism,^[15,16]^ resulting in a comparatively low required dose. According to a dose-escalation phase I study conducted at West China Hospital, a 200 to300 mg oral dose of donafenib twice-daily is recommended.^[10]^ An open-label, randomized phase II/III trial showed that donafenib (200 mg twice-daily) had a better efficacy than sorafenib (400 mg twice-daily) in patients with unresectable or metastatic HCC.^[17]^ Moreover, because of the lower dosage, there were fewer patients with serious adverse effects in the donafenib group than in the sorafenib group. Presently, donafenib is approved for the treatment of advanced HCC in China. Clinical trials are being conducted on its use in colorectal cancer, thyroid cancer, and other solid tumors. In addition, trials on combination treatment with PD-1 and PD-L1 inhibitors are ongoing.

In summary, we present the case of a patient diagnosed with advanced HCC, in whom the tumor mass was successfully controlled with donafenib. Donafenib is the second approved deuterium-containing drug and is a promising targeted drug for advanced HCC. Ongoing trials of donafenib and other deuterium derivatives will reveal new uses of these targeted drugs as well as additional information about them.

## 4 Patient's perspective

Taking donorafenib did not feel too bad. I felt just a little abdominal distension. There was no obvious discomfort. Blood examinations revealed no abnormalities. The disease was controlled after administering this targeted medicine. Therefore, I hope that more people will know about this new drug and be provided with more treatment options.

## Author contributions

Manuscript concepts and design: Qiaoqi Li, Hong Zhu Manuscript preparation: Qiaoqi Li Manuscript editing: Qiaoqi Li, Hong Zhu

Drafting of manuscript and/or critical revision: Qiaoqi Li, Hong Zhu

Approval of final version of manuscript: Hong Zhu

**Resources:** Hong Zhu.

**Writing – original draft:** Qiaoqi Li.

**Writing – review & editing:** Hong Zhu.
